# Impulsivity in remitted bipolar disorder

**DOI:** 10.1192/j.eurpsy.2021.516

**Published:** 2021-08-13

**Authors:** I. Lajmi, N. Smaoui, K. Yaich, R. Feki, S. Omri, M. Maalej Bouali, L. Zouari, J. Ben Thabet, N. Charfi, M. Maalej

**Affiliations:** Psychiatry C Department, Hedi chaker university hospital, sfax, Tunisia

**Keywords:** bipolar disorder, Impulsivity

## Abstract

**Introduction:**

Bipolar disorders (BD) are associated with a high degree of impulsivity especially during manic and depressive episodes. However, there is little information on impulsivity during remission phase.

**Objectives:**

Our objective was to assess impulsivity in patients with BD in remission compared to healthy controls (HC).

**Methods:**

This was a comparative, cross-sectional and analytical study, conducted in the outpatient psychiatry department of Hedi Chaker University Hospital in Sfax (Tunisia), from July to September 2019, among 30 patients with BD in remission compared to 34 HC. Data were collected on a pre-established questionnaire. Impulsivity was assessed with the Barratt Impulsiveness Scale (BIS-11).

**Results:**

Mean ages of BD patients and HC were 44.17 and 40.1 years, respectively. The sex ratio was 1.7 in BD patients and 0.9 in HC groups. Age of onset of BD was 30 years. Impulsivity scores of the BD patients were higher than HC on total (66.27 vs 53.53) and three subscales measures; motor (21.83 vs 16.15), attentional (15.83 vs 13.53) and non planning impulsivity (28.93 vs 23.71). High degree of impulsivity was noted in 33.3% of BD patients. BD patients scored significantly higher than the HC on total, motor, and non planning impulsivity scores (p = 0.001; p = 0.001; p=0.000, respectively)

**Conclusions:**

Our study found that patients with BD had a high degree of impulsivity outside the critical period compared to healthy individuals. Would this impulsivity be a vulnerability marker to the risk of early onset of the disease or the risk of its relapse?

